# Immunological reactions by T cell and regulation of crucial genes in treated celiac disease patients 

**Published:** 2020

**Authors:** Mohammad Rostami-Nejad, Zahra Razzaghi, Somayeh Esmaeili, Sina Rezaei-Tavirani, Alireza Akbarzadeh Baghban, Reza Vafaee

**Affiliations:** 1 *Gastroenterology and Liver Diseases Research Center, Research Institute for Gastroenterology and Liver Diseases, Shahid Beheshti University of Medical Sciences, Tehran, Iran*; 2 *Laser Application in Medical Sciences Research Center, Shahid Beheshti University of Medical Sciences, Tehran, Iran*; 3 *Traditional Medicine and Material Medical Research Center, Department of Traditional Pharmacy, School of Traditional Medicine,, Shahid Beheshti University of Medical Sciences, Tehran, Iran*; 4 *Basic and Molecular Epidemiology of Gastrointestinal Disorders Research Center, Research Institute for Gastroenterology and Liver Diseases, Shahid Beheshti University of Medical Sciences, Tehran, Iran*; 5 *Proteomics Research Center, School of Rehabilitation, Shahid Beheshti University of Medical Sciences, Tehran, Iran *; 6 *Proteomics Research Center, Shahid Beheshti University of Medical Sciences, Tehran, Iran *

**Keywords:** Celiac disease, Gene expression, Network analysis, Immune system

## Abstract

**Aim:**

To assess the immunological reactions and gene expression level in the celiac disease (CD) patients under a gluten-free diet (GFD).

**Background:**

CD is an autoimmune disorder in genetic susceptible individuals and lifelong gluten free diet is the effective treatment method. It seems that treated patients will experience a normal life style though there are documents about some potential damages.

**Methods:**

Gene expression profiles of treated CD patients and healthy samples were obtained from Gene Expression Omnibus (GEO) and compared to find the differentially expressed genes (DEGs). The identified DEGs were introduced in the network and gene ontology (GO) analysis.

**Results:**

Ten differentially expressed genes (DEGs) including CCR2, IRF4, FASLG, CCR4, ICOS, TNFSF18, BACH2, LTF, PRM1, and PRM2 were investigated via network analysis. Seven clusters of biological processes (BP) were determined as the affected BP. PThe finding led to introduction of CCR2, IRF4, FASLG, CCR4, and ICOS as the potential immunological markers that are still active despite GFD in the treated CD patients.

**Conclusion:**

The results of this study indicated that the immune system is already active in treated CD patients despite GFD treatment and exposure to gluten causes potential immunological reactions in these patients.

## Introduction

 Celiac disease (CD), as an autoimmune disorder, is a disease which appears in sensing and immune reaction responses to gluten. It has been confirmed that both genetic and environmental factors are involved in promotion of CD. Different extra- and intra-GI symptoms such as iron deficiency anemia, osteoporosis, weight loss, diarrhea, bloating etc. are predominant in CD patients (1, 2). Initial serological screening (tTg IgA/IgG, EMA IgA and DGP IgA/IgG) and biopsy of small intestine are the two well-known diagnostic methods which are used in the clinical practice with a respectable efficiency (3). Fathi et al. introduced lactate, valine, and lipid metabolites of serum as differential factors that discriminate between CD patients and healthy human (4). Rostami-Nejad, M et al. reported that IL2, PIK3CA, PRDM10, AKT1, and SRC as differentially expressed genes in intraepithelial Lymphocytes separating CD samples from controls. Based on this document, CD4+, CD25+, and alpha-beta regulatory T cell differentiation are the prominent biological markers dysregulates in the CD samples (5). 

Network analysis is an attractive method to assess different kinds of diseases. In this approach, the differentially expressed proteins, metabolites, or genes interact to form an interactome unit. Topological parameters of the constructed network are assessed and the critical ones will be introduced. The reported critical biomolecules can be candidate as biomarkers (6-8). Degree as a centrality parameter, which corresponds to the numbers of connections between an element of the network with the other nodes, is frequently used to characterize the crucial nodes of the studied networks (9, 10). 

Gene ontology is the other common approach applied to assess molecular function, biological processes, cellular components, and biochemical pathways related to the studied genes or proteins (11-14). Biological process analysis provides useful information about the roles of studied proteins in the body (15). In this study, gene expression profiles of treated celiac patients and healthy humans were compared via network analysis to see whether immunological reactions are still active after 2 years GFD or not in the treated CD patients. 

## Methods

GSE61849/GPL19242 (https://www.ncbi.nlm.nih.gov/geo/query/acc.cgi?acc=gse61849) was extracted from GEO. GSM1515692-706 and GSM1515707-21 were determined as treated celiac patients and healthy controls, respectively. The statistically distribution of gene expression profiles was assessed via box plot analysis. Certain GSMs which were not matched via box plot analysis were removed for more investigations. The significant characterized DEGs were identified regarding p-value≤0.01 and fold change≥1.5. Genes’ IDs were searched in Termofisher.com to find symbols of genes. 

The introduced significant DEGs were included in PPI network via STRING database with the network constructed by Cytoscape software (16). The queried genes interacted via a limited numbers of connections with no considerable information not obtained; thus to make an interactome, 20 first neighbors were added to the queried DEGs. The network was analyzed via “Network analyzer” and the nodes were visualized based on degree value. The 30 DEGs and neighbors were enriched to find the related biological processes via ClueGO plugin of Cytoscape software (17). The biological terms were clustered based on p-value where similar significant biological processes were grouped together. For better understanding, the terms which were not related to the queried DEGs were not considered for further assessment. 

## Results

**Figure 1:**
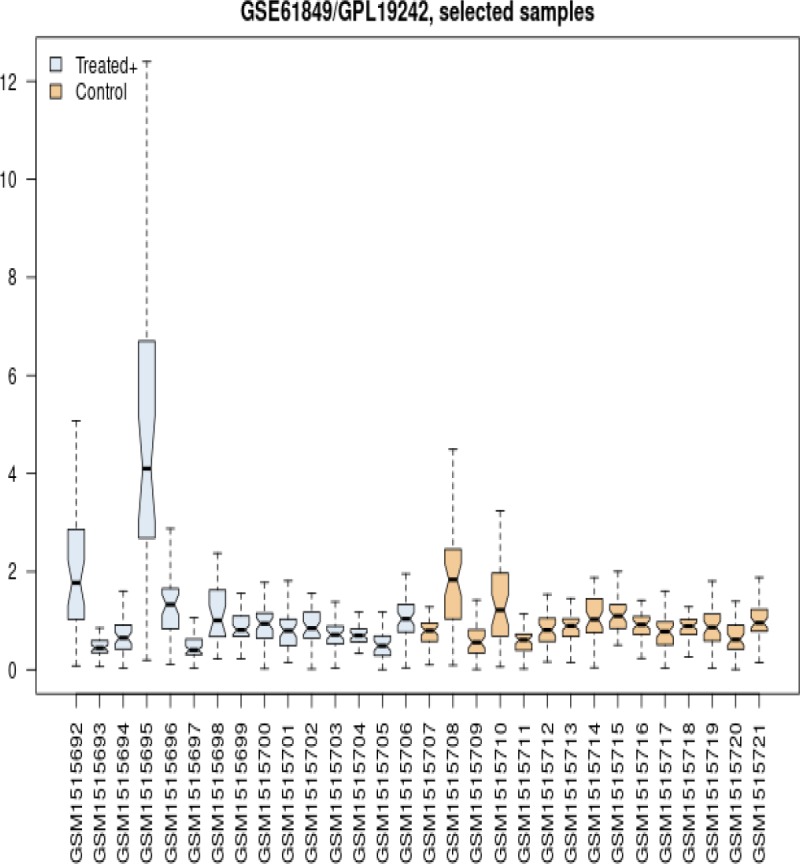
Box plot analysis of distribution of 15 gene expression profiles of healthy samples as control group and 15 celiac treated patients

Figure 1 compares the distribution of 15 genes expression profiles of healthy samples as control group with 15 celiac treated patients via box plot analysis. There are two control samples along with two treated patient gene expression profiles that are not consisted with statistical criteria.

**Figure 2:**
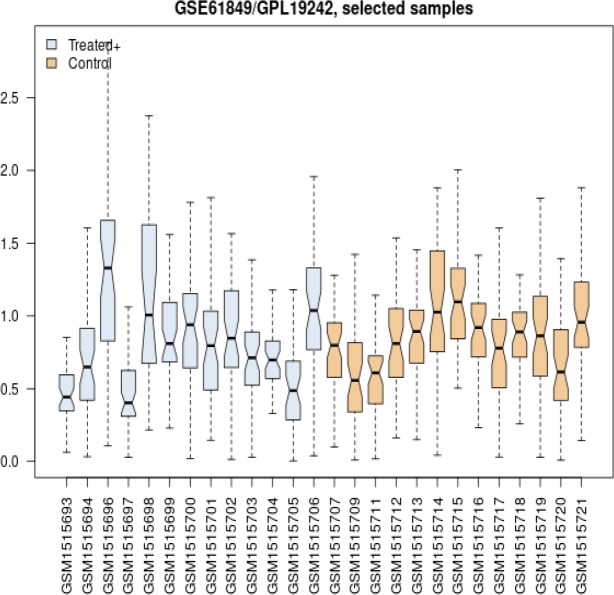
Box plot analysis of distribution of 13 gene expression profiles of healthy samples as control group and 13 celiac treated patients

Again, the profiles were compared without the mentioned four samples and data were almost comparable (see Figure 2). Figure 2 has been adjusted statistically, where there are fluctuations between profiles of samples obtained from limited amounts of vertical axis. Finally, the gene expression profiles of control and samples without the omitted 4 median uncentered profiles where candidate to be further investigated. 

**Figure 3:**
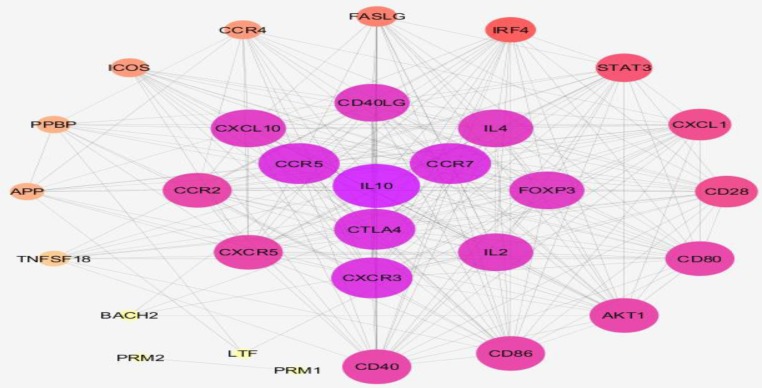
A network including the 10 queried DEGs and the 20 first neighbors. The nodes have been laid out based on degree value. Confidence score=0.4 as the default value of software was regarded

**Figure 4:**
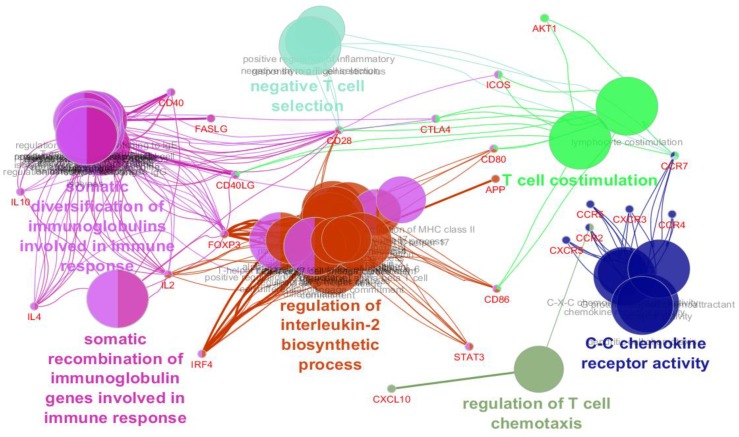
Biological processes related to the elements of the constructed network extracted from GO_BiologicalProcess-EBI-UniProt-GOA_27.02.2019_00h00. Names of clusters are highlighted. P-value and group P-value were considered less than 0.01

Ten DEGs including CCR2, IRF4, FASLG, CCR4, ICOS, TNFSF18, BACH2, LTF, PRM1, and PRM2 were identified as significant and characterized DEGs based on the comparison between treated celiac patients and control gene expression profiles. The selected DEGs were imported to the protein query of STRING database and the network was constructed by Cytoscape software. Due to poor interaction between the DEGs, 20 first neighbor genes were added to the queried individuals and a new network was created. As presented in Figure 3, the network was analyzed and laid out based on the degree value. Seven clusters of biological processes related to the queried DEGs and their 20 first neighbors were determined as displayed in Figure 4. For better understanding, the biological processes were screened to find the role of the queried DEGs; only the biological terms associated with the DEGs were selected while the other terms were disregarded (see Table 1).

## Discussion

There are limited approaches to the treatment of CD patients. A firm long-life gluten free diet is the only current treatment for the CD (18). Widespread efforts are focused on finding new therapeutically methods based on food processes, enzymatic procedures, modulation of immune system, and other techniques (19). Here, the possible latent aspects of CD in the treated patients have been evaluated. 

**Table 1:** Clusters of biological terms that are related to the queried DEGs. Names of groups are highlighted

GO Term	Group	Associated Genes
regulation of T cell chemotaxis	1	CCR2
lymphocyte co-stimulation	2	ICOS
T cell co-stimulation		ICOS
dendritic cell chemotaxis	3	CCR2, CCR4
G protein-coupled chemoattractant receptor activity		CCR2, CCR4
chemokine receptor activity		CCR2, CCR4
C-C chemokine receptor activity		CCR2, CCR4
positive regulation of endothelial cell apoptotic process	4	FASLG
somatic recombination of immunoglobulin genes involved in immune response*		
regulation of T-helper cell differentiation	5	IRF4
T-helper cell lineage commitment		IRF4
T-helper 17 cell differentiation		IRF4
T-helper 17 cell lineage commitment		IRF4
interleukin-2 biosynthetic process		IRF4
interleukin-4 biosynthetic process		IRF4
regulation of interleukin-2 biosynthetic process		IRF4
regulation of interleukin-4 biosynthetic process		IRF4
positive regulation of interleukin-2 biosynthetic process		IRF4
positive regulation of interleukin-4 biosynthetic process		IRF4
T cell lineage commitment		IRF4
alpha-beta T cell lineage commitment		IRF4
CD4-positive or CD8-positive, alpha-beta T cell lineage commitment		IRF4
CD4-positive, alpha-beta T cell lineage commitment		IRF4
regulation of T-helper cell differentiation	6	IRF4
T-helper 17 cell differentiation		IRF4
T cell tolerance induction		ICOS
somatic diversification of immunoglobulins involved in immune response		

*; somatic recombination of immunoglobulin genes involved in immune response was related to the first neighbor node.

A part of published data from Plaza-Izurieta L et al. (20) related to the treated CD patients and healthy individuals, was analyzed (see figures 1 and 2) to find the potential damage factors in the treated CD patients.

As depicted in Figure 3, 10 DEGs are connected to each other and are differentiated by the first neighbor nodes in interactom. Based on top degree value, CCR2, IRF4, FASLG, CCR4, ICOS, TNFSF18, BACH2, LTF, PRM1, and PRM2 are arranged in the network. Biological process analysis (Figure 4) revealed that not only the queried DEGs but also the first neighbor genes are associated with the biological processes involved in the immune system.

The finding showed that CCR2, IRF4, FASLG, CCR4, and ICOS, the five top nodes based on degree value, are related to the relevant biological processes. The other five DEGs were not involved in any biological processes possibly because of limitation of database content. As shown in Table 1, IRF4, CCR2, CCR4, ICOS, and FASLG are related to the 16, 5, 4, 3, and 1 biological processes respectively. Six clusters among the 7 introduced clusters, including 27 biological processes, are related to 50% of the queried DEGs. Both “regulation of T cell chemotaxis” and “T cell co-stimulation” are directly related to the T cells. Cluster 6 also refers to the substantial role of T cells in response to the CD in patients. Regulatory T cells play a critical role in maintaining peripheral tolerance. Investigations have shown that regulatory T cells deficiency is related to the pathogenesis of CD as well as the CD-associated autoimmunity (21). Considerable parts of biological processes in cluster 5 are related to the T-helper cells. The role of T-helper cell type 1 in the promotion of CD has been examined in several studies. Their findings showed that Il21 regulates this type of T-cells to produce cytokines in response to CD (22, 23). Romaldind Ceres C. et al. reported that the level of serum soluble interleukin 2 receptor is elevated in CD patients relative to the healthy children and untreated patients (24). As observed in Table 1, regulation of interleukin 2 biosynthesis is highlighted in terms of cluster 5 of biological processes. R Troncone et al. reported the role of interleukin 4 dysregulation in the CD patients (25). As with IL2, IL4 has appeared among biological processes of cluster 5. The other classes of biological processes refer to the involvement of immune responses in CD which is well-known correlation. It can be concluded that dysregulation of C-C chemokine receptor type 2 (CCR2), C-C chemokine receptor type 4 (CCR4), interferon regulatory factor 4 (IRF4), Fas ligand FASLG, and Inducible T-cell costimulatory (ICOS) signaling a deficiency in the immune system function, is the main feature of molecular events in treated patients. 

In conclusion, this study showed that, despite symptom and biological improvement through long-time GFD treatment in treated celiac patients, several immunological pathways are still active in this patients and exposure to the gluten may cause constant small intestine damages and increase the serum level titer of immunological markers.
